
JOURNAL INFORMATION
==============================
NLM Title Abbreviation: Biospektrum (Heidelb)
Journal ID: Biospektrum (Heidelb)
NLM Journal ID: 9615685

Biospektrum

ISSN: 0947-0867
EISSN: 1868-6249

ARTICLE INFORMATION
==============================
PMCID: PMC7556600
PMID: 33078045
DOI: 10.1007/s12268-020-1458-3
Article ID: 1458
Article version: 1
Subjects: Wissenschaft Methoden

Schnellnachweis von SARS-CoV-2 mit recombinase polymerase amplification

Behrmann Ole ole.behrmann@mhb-fontane.de http://www.mhb-fontane.de/einrichtung-details/institut-fuer-mikrobiologie-und-virologie-virologie.html
1 2
Bachmann Iris 1
Hufert Frank 1
Dame Gregory 1
1 grid.473452.3 Institut für Mikrobiologie und Virologie, Medizinische Hochschule Brandenburg Theodor Fontane (MHB), Universitätsplatz 1, D-01968 Senftenberg, Deutschland
2 grid.5963.9 Professur für Sensoren, Institut für Mikrosystemtechnik (IMTEK), Universität, Freiburg, Deutschland
Electronic publication date: 2020 Oct 14
Print publication date: 2020
Volume: 26
Issue: 6
First page: 624
Last page: 627
Copyright: © Die Autoren 2020
License: Open Access Dieser Artikel wird unter der Creative Commons Namensnennung 4.0 International Lizenz veröffentlicht, welche die Nutzung, Vervielfältigung, Bearbeitung, Verbreitung und Wiedergabe in jeglichem Medium und Format erlaubt, sofern Sie den/die ursprünglichen Autor(en) und die Quelle ordnungsgemäß nennen, einen Link zur Creative Commons Lizenz beifügen und angeben, ob Änderungen vorgenommen wurden. Die in diesem Artikel enthaltenen Bilder und sonstiges Drittmaterial unterliegen ebenfalls der genannten Creative Commons Lizenz, sofern sich aus der Abbildungslegende nichts anderes ergibt. Sofern das betreffende Material nicht unter der genannten Creative Commons Lizenz steht und die betreffende Handlung nicht nach gesetzlichen Vorschriften erlaubt ist, ist für die oben aufgeführten Weiterverwendungen des Materials die Einwilligung des jeweiligen Rechteinhabers einzuholen. Weitere Details zur Lizenz entnehmen Sie bitte der Lizenzinformation auf http://creativecommons.org/licenses/by/4.0/deed.de.
License URL: https://creativecommons.org/licenses/by/4.0/

issue-copyright-statement: © Springer-Verlag GmbH Deutschland, ein Teil von Springer Nature 2020

==============================
The COVID-19 pandemic highlights the need for fast and simple assays for nucleic acid detection. As an isothermal alternative to RT-qPCR, we outline the development of a detection scheme for SARS-CoV-2 RNA based on reverse transcription recombinase polymerase amplification (RT-RPA) technology. RPA uses recombination proteins in combination with a DNA polymerase for rapid amplification of target DNA at a constant temperature (39–42 °C) within 10 to 20 minutes and can be monitored in real-time with fluorescent probes.

■ Die derzeitigen Protokolle zur Diagnose von SARS-CoV-2-Infektionen beruhen auf der quantitativen reversen Transkriptions-PCR (RT-qPCR) für den Direktnachweis der viralen RNA [1]. PCR-basierte Methoden sind jedoch aufgrund des notwendigen energieintensiven Thermocyclings für schnelles und dezentrales Screening vor Ort schwierig umsetzbar. Alternativ sind seit einigen Jahren isotherme Verfahren – wie die *loop-mediated isothermal amplification* (LAMP) [2] oder die *recombinase polymerase amplification* (RPA) [3] – verfügbar, welche der PCR hinsichtlich Sensitivität und Spezifität gleichwertig sind. Diese Methoden erfordern keine hochentwickelten Thermocycling-Instrumente und beruhen auf enzymatischen Prozessen. Aufgrund ihres vergleichsweise einfachen Designansatzes, milder Prozesstemperatur (39–42 °C), einer rasanten Amplifikationsgeschwindigkeit (10–20 min.) und der Verfügbarkeit von Reagenzien in lyophilisierter Form sehen wir die RPA als vielversprechende isotherme Nukleinsäure-Nachweismethode für die Vor-Ort-Diagnostik (Point-of-Care-Testing, POCT) an. Aufbauend auf vorhergehenden Arbeiten, in welchen der Nachweis anderer Coronaviren wie MERS-CoV [4] und Bovines Coronavirus (BCoV) [5] mittels RPA demonstriert wurde, stellen wir in diesem Artikel die Entwicklung eines RPA-Assays für den Schnellnachweis von SARS-CoV-2 vor. Zusätzlich zum eigentlichen Assaydesign präsentieren wir auch einen neuartigen Ansatz für die Konstruktion von fluoreszenzmarkierten Oligonukleotidsonden für den Echtzeitnachweis der RPA.

## Funktionsprinzip der *recombinase polymerase amplification* (RPA)

Das grundlegende Funktionsprinzip der RPA ist dem natürlichen Replikationsmechanismus von Bakteriophage T4 nachempfunden (**Abb. 1**). Im ersten Schritt bindet das Enzym T4 UvsX (*recombinase*) mit T4 UvsY (*loading factor*) als Koenzym an die Primer. Dieser Nukleoproteinkomplex bindet unter Ausbildung einer Verdrängungsschleife (D-Loop, in **Abb. 1C** angedeutet) an die zum Primer komplementäre doppelsträngige Ziel-DNA. Nach der Hybridisierung löst sich UvsX vom Primer, während die geöffnete Verdrängungsschleife durch einzelstrangbindenende Proteine (T4 gp32) stabilisiert wird. Nun wird das freie 3'-Ende des Primers von einer mesophilen DNA-Polymerase mit Strangverdrängungsaktivität, z. B. Bsu oder Sau, verlängert. Dieser Prozess wird sowohl vom Vorwärts- als auch vom Rückwärtsprimer gleichzeitig initiiert, was zu einem exponentiellen Kopierprozess der Ziel-DNA führt.

Als weitere Komponenten sind dNTPs als Bausteine für die DNA-Synthese sowie ATP als Energiequelle und hochmolekulares PEG zur Steigerung der Reaktivität im RPA-Mix enthalten. Durch Hinzufügen einer reversen Transkriptase (RT) kann die RPA-Reaktion auch zum Nachweis von RNA verwendet werden. Hierfür erfolgt zunächst die Synthese komplementärer DNA (cDNA) ausgehend von dem zur Ziel-RNA komplementären Primer. Die so entstandene komplementäre DNA wird sofort vom eigentlichen RPA-Mechanismus erkannt und, wie zuvor beschrieben, kopiert. Die für die RPA verwendeten Primer sind mit 30 nt bis 35 nt länger als typische PCR-Primer, da nicht die Schmelztemperatur, sondern die Bindungsfähigkeit an UvsX für die Funktionalität bestimmend ist [6].

## Sondensysteme für die RPA

Da der RPA-Prozess in vielen Fällen neben dem gewünschten Produkt weitere Nebenprodukte erzeugt, ist ein Echtzeitnachweis der RPA durch interkalierende Fluoreszenzfarbstoffe, wie z. B. SYBR Green, nicht empfehlenswert. Für den fluoreszenzbasierten Nachweis stehen exo-Sonden zur Verfügung. Hierbei handelt es sich um zum gewünschten RPA-Produkt komplementäre einzelsträngige DNA-Oligonukleotide mit einer Länge von mindestens 46 nt. Intern ist das Oligonukleotid mit einer Struktur bestehend aus einem Fluorophor, einer fehlenden Base (AP-Stelle) sowie einem Fluoreszenzquencher modifiziert (**Abb. 2**). Hybridisiert dieses Sondenmolekül an das komplementäre RPA-Produkt, wird die nun im Doppelstrang enthaltene AP-Stelle von der AP-Endonukleaseaktivität der Exonuklease III (ExoIII) erkannt und gespalten. Dies führt zu einer räumlichen Trennung von Fluorophor und Quencher, wodurch ein zur DNA-Kopienzahl proportionales Fluoreszenzsignal entsteht. Zudem dient das nun freigewordene 3'-Ende als weiterer Primer und wird von der DNA-Polymerase verlängert.

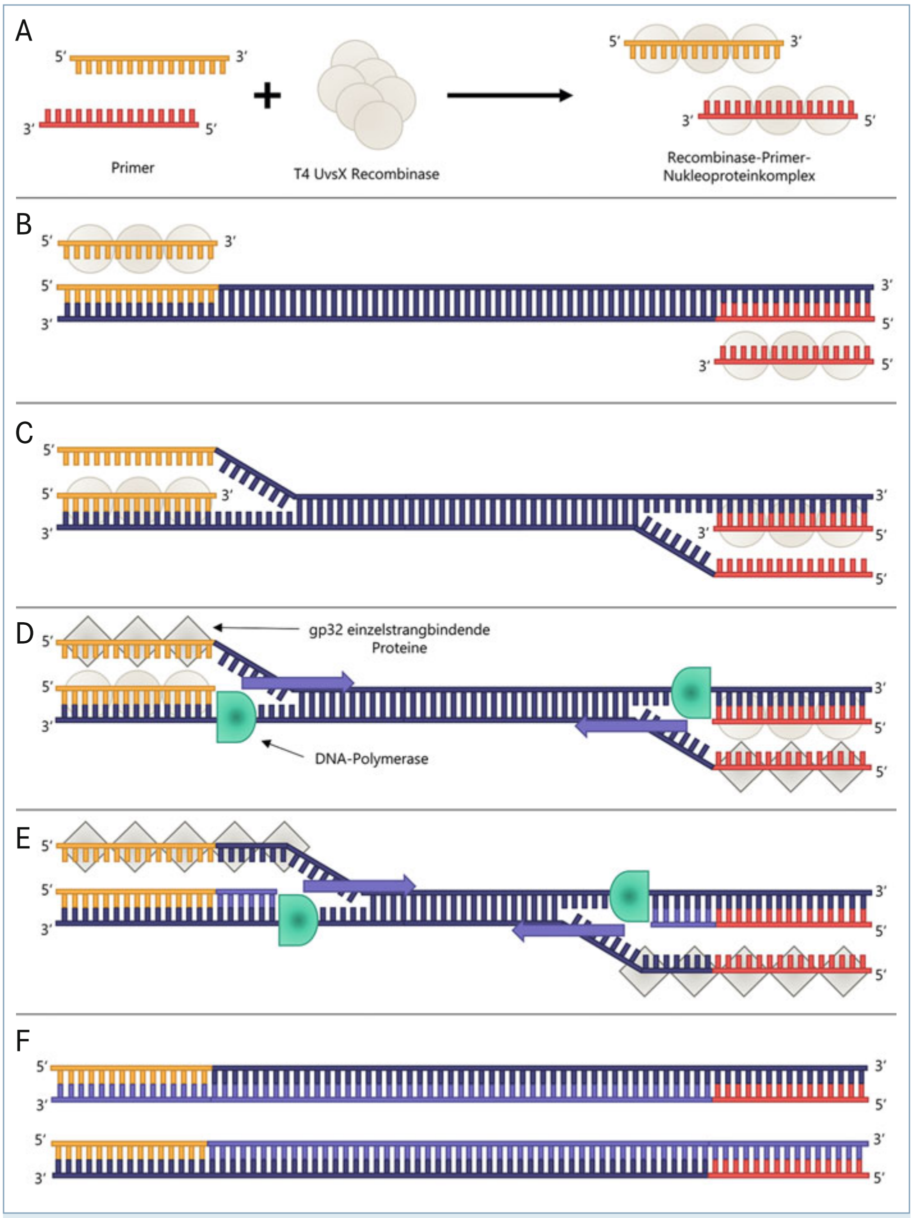

▲ **Abb. 1:** Prinzip der *recombinase polymerase amplification* (RPA). **A**, Bildung des Nukleoproteinkomplexes. **B**, Die Nukleoproteinkomplexe finden ihre Komplementärsequenzen in der Ziel-DNA. **C**, Anbindung der Primer unter Ausbildung einer Verdrängungsschleife. **D**, Stabilisierung der Verdrängungsschleife durch gp32 und Anlagerung der DNA-Polymerase. **E**, Verlängerung der Primer durch DNA-Polymerase. **F**, Die Zielsequenz wurde kopiert.

## Herausforderungen für das Sondendesign

Eine Herausforderung im Design von Sonden nach dem exo-Prinzip ist der interne Einbau von Fluorophor- und Quenchermolekülen. Diese sind kommerziell zu einem vertretbaren Preis nur als an Thymin gebundene Bausteine verfügbar. Da ein ausreichendes Quenching nur dann gegeben ist, wenn Fluorophor und Quencher nicht weiter als ein bis fünf Nukleotide voneinander entfernt sind, muss die Sondensequenz zwei Thymine in diesem Abstand aufweisen. Im Zusammenhang mit weiteren Designregeln bezüglich Homo- und Heterodimerbildung von exo-Sonde und Primern führt dieses oft zu Einschränkungen in der Wahl der Zielsequenz [7]. Unsere Arbeitsgruppe konnte jedoch kürzlich mit der Entwicklung des exo-IQ (*internally quenched*)-Sondenprinzips eine Lösung für dieses Problem präsentieren [8]. Durch Verwendung einer alternativen, kommerziell verfügbaren Anbindungsmethode für das Quenchermolekül über einen Zwischenlinker (**Abb. 2**) kann dieses unabhängig von der Basenabfolge der Sondensequenz eingebaut werden. Dadurch ist lediglich das Vorhandensein eines Thymins notwendig. Durch die so gewonnene Flexibilität in der Auswahl der Sondensequenz kann die exo-IQ-Sonde nach einem festen Schema erstellt werden. Hierdurch ist zudem gewährleistet, dass sich Fluorophor und Quencher immer im geringen Abstand von nur zwei Basen positionieren lassen, was eine deutlich bessere Funktion der Sonde durch ein höheres Signal-Rausch-Verhältnis wahrscheinlicher macht.

## Design eines RPA-exo-IQ-Assays am Beispiel von SARS-CoV-2

Das für den Virusnachweis gewählte Zielgen muss hochspezifisch sein und einer geringen Mutationswahrscheinlichkeit unterliegen sowie vorzugsweise etwa 1 kb lang sein, um genügend Flexibilität in der Positionierung der RPA-Primer und exo-IQ-Sonde zu gewährleisten. Im Fall der Coronaviren bietet sich hierfür das Nukleoprotein-Gen (N-Gen) mit einer Länge von etwa 1,2 kb an, da dieses zusätzlich während der viralen Replikation auch in sehr hoher Kopienzahl vorliegt, wodurch die diagnostische Sensitivität erhöht werden kann. Geeignete RPA-Primer- und Sondensequenzen konnten bisher lediglich semi-automatisch unter Zuhilfenahme von angepasster PCR-Designsoftware erstellt werden. Seit 2019 kann dies jedoch mit *PrimedRPA* [9] erfolgen, der ersten Designsoftware speziell für RPA-exo-Assays. Ausgehend von diesen Daten werden zunächst alle Sonden ausgewählt, welche ein Thymin an Position 30 besitzen und die gewählten Sequenzen werden mittels Alignment auf ihre Selektivität bezüglich der gewünschten Zielsequenz geprüft. Bei der Entwicklung einer SARS-CoV-2-RPA muss hier insbesondere auf die Selektivität gegenüber SARS-CoV sowie weiterer respiratorischer Viren, welche ein ähnliches klinisches Bild hervorrufen, geachtet werden. Da der Nachweis von SARS-CoV-2 aus Nasen-/Rachenabstrichen erfolgen soll, muss zusätzlich eine Kreuzreaktivität mit humaner DNA ausgeschlossen sein. Ist eine Sondensequenz gefunden, die alle theoretischen Bedingungen erfüllt, werden zwei bis vier Paare flankierender Primer aus der von *PrimedRPA* generierten Liste ausgewählt. Nach der Synthese der Primerpaare und der Sonde wird jede mögliche Kombination aus Vorwärts- und Rückwärtsprimer in Kombination mit der Sonde getestet. Als Zielsequenz wird ein *in vitro*-Transkript des SARS-CoV-2 N-Gens

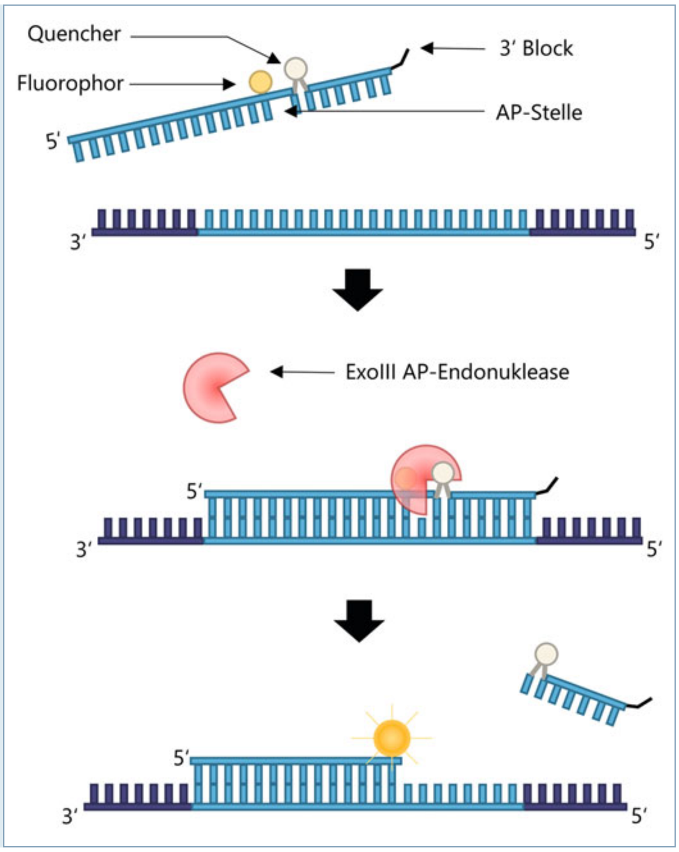

▶ **Abb. 2:** Struktur und Funktionsprinzip der RPA exo-IQ-Sonde. Im ersten Schritt hybridisiert die Sonde an ihre komplementäre Zielsequenz. Die ExoIII erkennt nun die im Doppelstrang vorliegende AP-Stelle und spaltet die Sonde, wodurch Fluorophor und Quencher getrennt werden. Dies führt zu einem zur Konzentration des RPA-Produkts proportionalen Anstieg der Fluoreszenz.

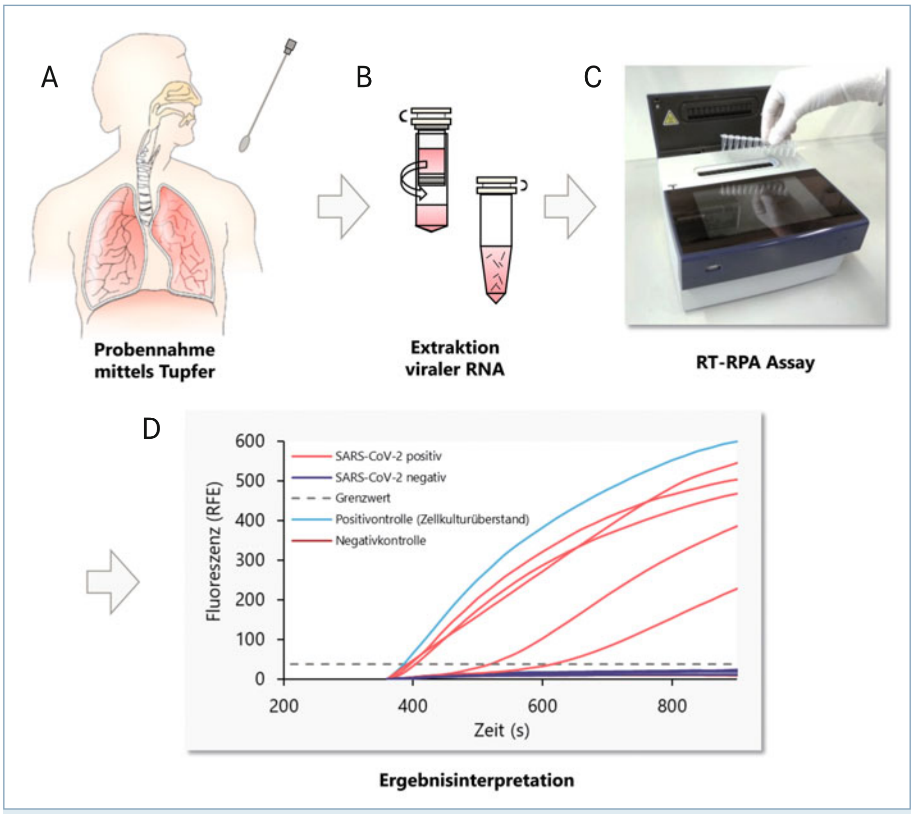

▲ **Abb. 3:** Ablaufschema des SARS-CoV-2-Schnellnachweises mittels RT-RPA. **A**, Probennahme mittels Nasen-/Rachenabstrich. **B**, Extraktion der SARS-CoV-2-RNA aus der Patientenprobe. **C**, Durchführung der SARS-CoV-2-spezifischen RT-RPA. **D**, Ergebnisinterpretation/Befundung anhand der Fluoreszenzsignalverläufe.

in definierter Konzentration verwendet. Die Primerpaare werden nun anhand ihrer Reaktionsgeschwindigkeit und Fluoreszenzintensität bewertet. Ist eine vielversprechende Kombination gefunden, wird diese mittels einer Verdünnungsreihe der Zielsequenz auf ihre Sensitivität geprüft. Konnte keine gute Kombination gefunden werden, werden weitere Primer ausgewählt oder bestehende in Länge und/oder Position verändert, bis ein zufriedenstellendes Ergebnis erreicht wird. Nach unserer Erfahrung ist die Wahl der RPA-Primer und nicht so sehr die exo-IQ-Sonde für die Funktionsfähigkeit eines Assays ausschlaggebend. Für die beste ermittelte Kombination aus Primern und Sonde muss nun die zuvor *in silico* erfolgte Selektivitätsbestimmung experimentell verifiziert werden. Hierfür wird der Assay mit Nukleinsäureproben aus Zellkultur anderer respiratorischer Viren durchgeführt. Wird keine Kreuzreaktivität beobachtet, kann der Assay mit vollständigen SARS-CoV-2-Virusgenomen aus Zellkultur analysiert und dann mit Patientenproben validiert werden (**Abb. 3**). Nach den Herstellervorgaben beträgt das Reaktionsvolumen eines RPA-Ansatzes 50 µl, wobei bis zu 5 µl Probe eingesetzt werden können. Da im Fall von SARS-CoV-2 eine sehr hohe Konzentration an Zielmolekülen in Patientenproben vorhanden ist, kann das Reaktionsvolumen auf ein Achtel reduziert werden. Dies ist insbesondere während Pandemien vorteilhaft, da es hier schnell zu Lieferengpässen von Reagenzien kommen kann.

## Ausblick

Nach der Entwicklung des RT-RPA-Assays steht nun die Integration in ein mobiles Gesamtsystem nach dem Vorbild eines portablen Kofferlabors [10] für den schnellen Vor-Ort-Einsatz an. Da eine traditionelle Probenpräparation über selektive Festphasenbindung für den mobilen Einsatz ungeeignet ist, sollen schnelle Einschritt-Präparationsverfahren, wie z. B. bereits von A. Ladha *et al.* [11] vorgestellt, auf ihre Kompatibilität mit der RPA untersucht werden. Zudem soll versucht werden, den bestehenden Assay zu einem Multiplex-Assay auszubauen. Hierfür soll der Assay um eine humane Extraktionskontrolle für die Bewertung der Probenqualität sowie weitere SARS-CoV-2-Zielgene erweitert werden. Zukünftig sind Projekte für den Schnellnachweis weiterer respiratorischer Erreger geplant.

## Danksagung

**Korrespondenzadressen:**

Ole Behrmann, M. Sc.
Institut für Mikrobiologie und Virologie
Medizinische Hochschule Brandenburg Theodor Fontane (MHB)
Universitätsplatz 1
D-01968 Senftenberg
ole.behrmann@mhb-fontane.de
www.mhb-fontane.de/einrichtung-details/institut-fuer-mikrobiologie-und-virologie-virologie.html

## AUTOREN

**Ole Behrmann**

**Iris Bachmann**

**Frank Hufert**

**Gregory Dame**


Funding

Open Access funding enabled and organized by Projekt DEAL.

Ole Behrmann 2006–2014 Studium der Mikrosystemtechnik an der Universität Freiburg. 2014–2018 Wissenschaftlicher Mitarbeiter an der Professur für Sensoren, IMTEK, Universität Freiburg. Seit 2016 Doktorand an der Universität Freiburg und der Medizinischen Hochschule Brandenburg. Seit 2018 Wissenschaftlicher Mitarbeiter am Institut für Mikrobiologie und Virologie, Medizinische Hochschule Brandenburg.

Iris Bachmann Seit 2013 Studium der Biotechnologie an der BTU Cottbus-Senftenberg. 2020 Masterarbeit am Institut für Mikrobiologie und Virologie, Medizinische Hochschule Brandenburg.

Frank Hufert 1979–1986 Studium Humanmedizin, Universität Hamburg, Approbation, 1988 Promotion ebenda. 1989 Dipl. Tropenmedizin, 1994 Facharzt für Mikrobiologie, Virologie und Infektionsepidemiologie. 1987–1992 Bernhard-Nocht-Institut, Assistentsarzt in den Abteilungen Virologie, Bakteriologie und in der klinischen Abteilung. 1992–2005 Institut für Medizinische Mikrobiologie und Hygiene, Abteilung Virologie, Universität Freiburg. 2005–2008 Professur und leitender Oberarzt, Abteilungsdirektor Virologie, ebenda. 2008–2014 Abteilungsdirektor Abteilung Virologie am Zentrum für Hygiene und Humangenetik, Universitätsmedizin Göttingen. Seit 2014 Institutsdirektor am Institut für Mikrobiologie und Virologie, Medizinische Hochschule Brandenburg.

Gregory Dame 1987–1993 Biologiestudium an der Universität Freiburg, 2003 Promotion ebenda. 2007–2015 Gruppenleiter am IMTEK, Universität Freiburg. Seit 2015 Laborleitung am Institut für Mikrobiologie und Virologie, Medizinische Hochschule Brandenburg.

Danksagung

Diese Arbeit wurde zum Teil durch das BMBF-Projekt „R & C.net“ (FKZ 01ZZ2051B) gefördert.


Literatur

[1] CDC (2020) 2019-Novel Coronavirus (2019-nCoV) Realtime rRT-PCR panel primers and probes. www.cdc.gov/coronavirus/2019-ncov/downloads/rt-pcr-panel-primer-probes.pdf
[2] Notomi T Loop-mediated isothermal amplification of DNA Nucleic Acids Res 2000 28 E63 10.1093/nar/28.12.e63 10871386 PMC102748
[3] Piepenburg O Williams CH Stemple DL DNA detection using recombination proteins PLoS Biol 2006 4 e204 10.1371/journal.pbio.0040204 16756388 PMC1475771
[4] Abd El Wahed A, Patel P, Heidenreich D et al. (2013) Reverse transcription recombinase polymerase amplification assay for the detection of middle East respiratory syndrome coronavirus. PLoS Curr 5:ecurrents.outbreaks. 62df1c7c75ffc96cd59034531e2e836410.1371/currents.outbreaks.62df1c7c75ffc96cd59034531e2e8364 PMC3871419 24459611
[5] Amer HM Abd El Wahed A Shalaby MA A new approach for diagnosis of bovine coronavirus using a reverse transcription recombinase polymerase amplification assay J Virol Methods 2013 193 337 340 10.1016/j.jviromet.2013.06.027 23811231 PMC7113639
[6] Li J Macdonald J Von Stetten F Review: a comprehensive summary of a decade development of the recombinase polymerase amplification Analyst 2019 144 31 67 10.1039/C8AN01621F 30426974
[7] TwistDx Ltd (2019) TwistAmp® DNA amplification kits assay design manual. www.twistdx.co.uk
[8] Behrmann O Bachmann I Spiegel M Rapid detection of SARS-CoV-2 by low volume real-time single tube reverse transcription recombinase polymerase amplification using an exo probe with an internally linked quencher (exo-IQ) Clin Chem 2020 66 1047 1054 10.1093/clinchem/hvaa116 32384153 PMC7239256
[9] Higgins M Ravenhall M Ward D PrimedRPA: Primer design for recombinase polymerase amplification assays Bioinformatics 2019 35 682 684 10.1093/bioinformatics/bty701 30101342 PMC6379019
[10] Abd El Wahed A Weidmann M Hufert FT Diagnostics-in-a-Suitcase: Development of a portable and rapid assay for the detection of the emerging avian influenza A (H7N9) virus J Clin Virol 2015 69 16 21 10.1016/j.jcv.2015.05.004 26209370 PMC7106543
[11] Ladha A, Joung J, Abudayyeh O et al. (2020) A 5-min RNA preparation method for COVID-19 detection with RT-qPCR. medRxiv preprint. www.medrxiv.org/content/10.1101/2020.05.07.20055947v1
